# Changes in the enzyme profile of the white-rot fungus *Bjerkandera adusta* in co-culture with the brown-rot fungus *Gloeophyllum trabeum* and its potential for dye decolorization

**DOI:** 10.1007/s11274-026-05018-5

**Published:** 2026-05-12

**Authors:** Bárbara Arias, Adriane Maria Ferreira Milagres, Angela Machuca

**Affiliations:** 1 Department of Biotechnology, Escola de Engenharia de Lorena, University of São Paulo, Lorena, CEP 12 602 810 SP Brazil; 2 Department of Plant Sciences and Technology, Escuela de Ciencias y Tecnologías, Universidad de Concepción, Los Ángeles, Chile

**Keywords:** Enzyme cocktails, Interspecific interactions, Ligninolytic enzymes, Peroxidases, RBB-R decolorization

## Abstract

Co-cultivation has emerged as an effective strategy to mimic natural microbial interactions and modulate extracellular enzyme production. In this study, interactions between the white-rot fungus *Bjerkandera adusta* and the brown-rot fungus *Gloeophyllum trabeum* were investigated with respect to growth and production of lignocellulolytic enzymes in solid and liquid cultures. Growth assays on solid medium revealed mixed mycelial growth without pigment formation at the interface zone, indicating partial compatibility between species. Co-cultivation led to pronounced changes in the enzyme activities compared to monocultures. Oxidative enzyme activities were strongly stimulated, with peroxidase activities increasing 2-3-fold relative to *B. adusta* monoculture, despite the absence of peroxidase in *G. trabeum*. Conversely, cellulolytic and hemicellulolytic activities were significantly reduced under co-culture conditions compared to *G. trabeum* monoculture. Zymogram analysis and ion-exchange chromatography confirmed that peroxidase production was restricted to *B. adusta* and the co-culture. The enzyme cocktail obtained from co-culture exhibited superior performance in Remazol Brilliant Blue R decolorization (∼ 90%) compared to *B. adusta* monoculture (∼40%). These findings demonstrate that interspecific fungal interactions can selectively shift the enzymatic balance toward oxidative mechanisms, highlighting cocultivation as a promising approach for generating enzyme systems with improved potential for dye bioremediation.

**Graphical abstracts:**

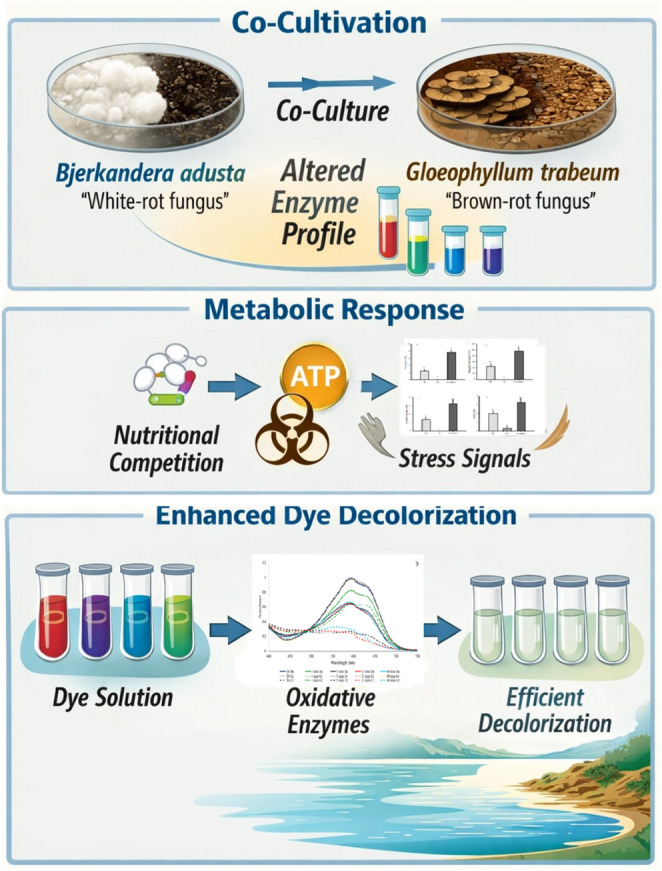

**Supplementary Information:**

The online version contains supplementary material available at 10.1007/s11274-026-05018-5.

## Introduction

Wood-decaying fungi are a diverse group of microorganisms capable of degrading lignocellulosic biomass through specialized extracellular enzyme systems. These organisms play a key ecological role in carbon cycling by decomposing plant biomass in natural environments and have gained attention for applications in biomass conversion and environmental remediation (Chen et al. 2022; Lin et al. 2022; Bari et al. 2024). Among them, white-rot fungi can degrade all major lignocellulose components, including lignin, through oxidative enzymes, whereas brown-rot fungi primarily depolymerize cellulose and hemicellulose while modifying lignin through non-enzymatic mechanisms. This functional diversity makes these organisms particularly attractive for the development of complementary enzymatic systems (Baghmare et al. 2025; Xun et al. 2025).

In natural environments, wood-decaying fungi rarely act in isolation, instead coexisting and interacting with other microorganisms during lignocellulosic colonization. However, most laboratory studies on lignocellulosic material degradation for biotechnological purposes generally employ monocultures, which restrict enzyme diversity and limit the full expression of metabolic potential (Soares et al. 2022). In this context, co-culture has emerged as a promising strategy to overcome genomic and metabolic constraints, enhance enzymatic expression, and complement catalytic capacities among microbial strains (Ming et al. 2019; Intasit et al. 2021). By cultivating fungi with distinct enzymatic repertoires, co-cultivation can generate enzyme cocktails with improved yields and broader functionalities.

Interactions within co-cultures may be antagonistic, resulting in growth inhibition, or synergistic, promoting mutual stimulation and activation of silent gene clusters (Peláez et al. 2022; Embacher et al. 2023; Kim 2023). Importantly, co-culture strategies are relatively simple and cost-effective and have been successfully applied to enhance enzyme expression and discover novel bioactive microbial products (Peng et al. 2021).

Interactions between wood-decaying fungi play a central role in lignocellulose decomposition dynamics. Combinations of brown-rot and white-rot fungi can alter degradation patterns compared to monocultures, influencing both enzymatic activity profiles and the chemical composition of decayed wood (Hiscox et al. 2015). Recent studies have demonstrated that co-cultivation of white-rot fungi, such as *Lenzites betulina* and *Trametes versicolor*, enhances lignin degradation through cooperative oxidoreductase activity, highlighting the importance of oxidative-hydrolytic interplay in overcome biomass recalcitrance (Cui et al. 2021). Consequently, there is growing interest in exploiting saprobic fungi to produce tailor-made enzyme cocktails as cost-effective alternatives to commercial formulations for lignocellulosic biomass conversion (Maleki et al. 2020).

In this context, the wood-decaying fungi *Bjerkandera adusta* and *Gloeophyllum trabeum* were selected as a model system. *B. adusta*, a white-rot fungus, selectively degrades lignin while preserving cellulose and is known for producing manganese peroxidases (MnP), laccases, and versatile peroxidases, enabling the oxidation of xenobiotic compounds, such as synthetic dyes (Heinfling et al. 1998; Korniłłowicz-Kowalska and Rybczyńska-Tkaczyk 2020; Gao et al. 2020; Sugano et al. 2021; Pinto et al. 2022). In contrast, *G. trabeum*, a brown-rot fungus, lacks class II peroxidases, but rapidly depolymerizes wood polysaccharides through Fenton chemistry and produces cellulases and hemicellulases for complete cell wall degradation (Goodell et al. 2020; Kojima et al. 2020; Rizqi et al. 2023). Their complementary degradative capacities of these fungi make their co-cultivation a compelling system for producing enzyme cocktails with diversified oxidative and hydrolytic functions (Sanitá-Lima and Coutinho de Lucas 2022; Rizqi et al. 2023).

Industrial effluents from textile, leather, and paper industries frequently contain synthetic dyes and xenobiotic compounds that are chemically stable, toxic, and resistant to conventional wastewater treatments (Gao et al. 2020). White-rot fungi, particularly *B. adusta*, have been widely explored for dye decolorization and detoxification through oxidative enzymes, while the hydrolytic activity of *G. trabeum* may further contribute to pollutant degradation. Co-cultivation of these fungi thus represents a promising strategy to expand enzymatic diversity and enhance the degradation of dyes, lignin, and related xenobiotics.

Therefore, this study investigates the impact of co-cultivating *B. adusta* and *G. trabeum* on lignocellulolytic enzyme profiles, providing insights into the potential of co-culture-derived enzyme cocktails for bioremediation applications, with particular emphasis on the decolorization of Remazol Brilliant Blue R (RBB-R).

## Materials and methods

### Fungal species

White- and brown-rot fungi were used in this study, namely *Bjerkandera adusta* (strain 30) and *Gloeophyllum trabeum* (ATCC 11539), respectively. *B. adusta* was obtained from the Fungal Biotechnology Laboratory culture collection (University of Concepción, Campus Los Ángeles, Chile) and *G. trabeum* from ATCC. Cultures were maintained on malt extract agar (MEA) at 25 °C in the dark for 10 days.

### Growth of fungal species in monoculture and co-culture on solid media

Petri dishes (100 mm) containing MEA and defined medium for *Trametes* (MDT) (Roy and Archibald 1993) were used. Co-culture plates were inoculated with a 5-mm agar/mycelium disc of *B. adusta* and *G. trabeum*, placed opposite each other, 5 cm apart and approximately 2 cm from the edge of the plate (Supplementary Fig. 1). Monoculture plates were inoculated with a single 5-mm agar/mycelium disc per strain, 2 cm from the edge. All inoculated plates were incubated at 25 °C in darkness for 10 days. Photographs were taken at 2 and 10 days of incubation, and the type of interaction in co-cultures was determined according to Mohammad et al. (2011) and Molla et al. (2001). At the end of the incubation period, colony diameters (cm) were measured. All assays were performed in duplicate.

### Qualitative enzyme assays in co-culture on solid medium

Detection of cellulolytic and ligninolytic activities in co-cultures was initially performed qualitatively on solid medium (MEA) supplemented with 1% carboxymethylcellulose (CMC) and 0.02% 2,2′-azino-bis (3-ethylbenzothiazoline-6-sulfonic acid) (ABTS), respectively. Plates were inoculated as described above and incubated at 25 °C for 7 days. After incubation, CMC plates were stained with 1% Congo Red solution for 30 min and washed with 1 M NaCl solution for 20 min (Khoirunnisa et al. 2020). Positive activity was indicated by the appearance of a hydrolysis halo around fungal colonies. For ABTS plates, positive activity was indicated by the development of green-blue/purple coloration in the medium due to ABTS oxidation (More et al. 2011).

### Submerged culture

Liquid MDT medium (pH 5) supplemented with 1% wheat bran was prepared for the quantitative detection of hydrolytic and ligninolytic enzymes in co-culture. Erlenmeyer flasks (250 mL) containing 100 mL of MDT and wheat bran were sterilized at 120 °C for 15 min. After reaching an appropriate temperature, the flasks were inoculated. Monocultures were inoculated with 4 agar discs (5 mm diameter) containing active mycelium of a single strain per flask. For co-cultures, 4 discs of each strain were added to the same flask (total of 8 discs per flask). All assays were performed in triplicate, and flasks were incubated at 25 °C and 120 rpm for 10 days.

After 10 days of incubation, culture broths from monocultures and co-cultures were filtered and concentrated fivefold by ultrafiltration (Amicon^®^ Stirred Cell, MilliporeSigma) using a 10 kDa membrane. Protein contents in concentrated extracts were determined according to Bradford (1976).

### Enzyme activity measurement

Endoglucanase (EG) activity was assayed using 0.44% (w/v) CMC (Tan et al. 1987), and xylanase activity using 1% (w/v) birchwood xylan (Bailey et al. 1992). Released reducing sugars were measured using the 3,5-dinitrosalicylic acid (DNS) method at 540 nm (Miller 1959). β-Glucosidase and β-xylosidase activities were measured using 0.1% (w/v) p-nitrophenyl-β-D-glucopyranoside (pNPG) and p-nitrophenyl-β-D-xylopyranoside (pNPX), respectively. Released p-nitrophenol was measured at 410 nm (Mallerman et al. 2015).

Peroxidase and laccase activities were determined by 5 mM ABTS oxidation in the presence and absence of 2 mM H_2_O_2_, respectively (Bourbonnais and Paice 1990). Manganese peroxidase (MnP) activity was assayed by oxidation of 0.1% phenol red in the presence of 2 mM H_2_O_2_ (Khindaria et al. 1994). Versatile peroxidase (VP) activity was determined using 20 mM 2,6-dimethoxyphenol (2,6-DMP) and 6 mM H_2_O_2_, in the presence and absence of manganese sulfate (MnSO_4_) (Mester and Field 1997), with VP activity expressed as the difference between activities with and without MnSO_4_. All assays were performed using a Hitachi U-2900 UV-Vis spectrophotometer. One enzyme unit (U) was defined as the amount of enzyme producing 1 µmol of product per minute.

### Purification and electrophoresis of extracellular proteins

Enzymatic extracts were purified by fast protein liquid chromatography (FPLC) using an Äkta Start/Cytiva system. Extracts were concentrated fivefold with a 10 kDa Amicon^®^ membrane prior to purification. Ion-exchange chromatography was performed using a 10 mL DEAE-Sepharose CL6B column equilibrated with 10 mM sodium phosphate buffer (pH 6.0). Proteins were eluted with a 0–500 mM NaCl gradient at 1 mL/min, collecting 1 mL fractions (Xiaobin et al. 2007). Endoglucanase and peroxidase activities were assayed in eluted fractions, and protein content was monitored at 280 nm to determine the protein elution profile and correlate it with enzymatic activities.

Protein profiles of concentrated fungal broths were evaluated by native polyacrylamide gel electrophoresis (PAGE). To estimate molecular weights and detect peroxidases via catalytic activity, gels were supplemented with 2 mM ABTS. Extracts were initially concentrated fivefold using a 10 kDa Amicon^®^ membrane and further concentrated 20-fold using a 30 kDa Microcon centrifugal filter unit. Gels were run in a Mini-PROTEAN Tetra Vertical Electrophoresis Cell at room temperature for 40–60 min at a constant 120 V.

After electrophoresis, the gel was split in two. One half was stained with Coomassie Blue for protein detection. The other half was used for a zymogram to visualize peroxidase activity by incubating it with 20 mM H_2_O_2_, pH 5.0, for 30 min at 50 °C. The appearance of dark green bands indicates a positive reaction to ABTS oxidation (Oloketuyi et al. 2020). Images of both gels were captured using a Gel Doc EZ system and Image Lab 5.2.1 software.

### Decolorization of remazol brilliant blue reactive (RBBR)

RBBR decolorization was evaluated at a final concentration of 100 mg/L using concentrated 5-fold cell culture broth from *G. trabeum* and *B. adusta* monocultures and co-cultures. Concentrated samples were standardized by total protein content (10 µg/ml), and for *B. adusta* and co-culture, also by peroxidase activity (0.02 U/ml), since *G. trabeum* does not produce oxidative enzymes.

Reactions were performed at 30 °C in darkness for different incubation times (0, 1, 5, 10, 15, 30, and 60 min) in 50 mM sodium acetate buffer (pH 5) with 2 mM H_2_O_2_ as cofactor. The absorption spectrum was measured from 400 to 750 nm to determine the maximum absorbance of the anthraquinone chromophore and to monitor decolorization kinetics.

The percentage of decolorization at λmax was calculated as:

$$\:Dye\:decolorization\:\left(Y,\%\right)=\frac{{Abs}_{\left(0\right)}-{Abs}_{\left(t\right)}\:}{{Abs}_{\left(0\right)}}*100$$

where Abs₀ and Abs_t_ correspond to the initial absorbance and the absorbance at time t, respectively. All experiments were performed in triplicate, and results are expressed as mean ± standard deviation (SD).

### Statistical analysis

Analysis of variance (ANOVA) was performed at a 95% confidence level to compare experimental data in triplicate using Statistica version 10. Tukey’s test was used to determine significant differences, with *P* ≤ 0.05 considered statistically significant.

## Results

### Mycelial Interaction between Bjerkandera adusta and Gloeophyllum trabeum on Solid Media

**Fig. 1:**
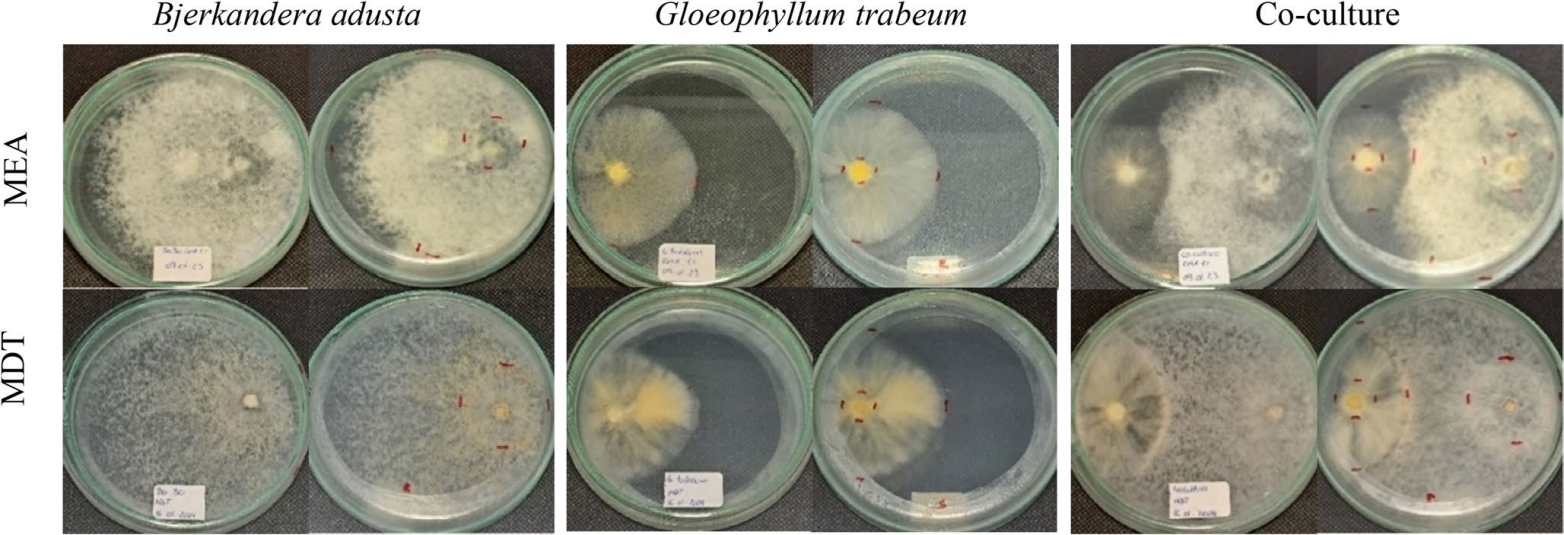
Growth pattern of *B. adusta* and *G. trabeum* in mono- and co-cultures on MEA and MDT plates (pH 5) after 10 days of incubation at 25 °C. In the co-cultures, the inoculum on the left corresponds to *G. trabeum* and the one on the right to *B. adusta*. Photographs of each fungus show the Petri dishes from the front (left) and the back (right)

Initially, the type of mycelial interaction between *B. adusta* and *G. trabeum* growing in mono- and co-culture was investigated in plates containing MEA and MDT solid media. In monoculture, *B. adusta* exhibited rapid and cottony growth, especially on MDT medium, fully covering the plate within 10 days. *G. trabeum* grew more slowly and produced less dense mycelium, showing a characteristic yellow coloration on MDT, with radial expansion approximately 50% lower than *B. adusta* (Fig. 1; Table 1). In co-culture, the radial growth of *B. adusta* was reduced compared to monoculture, particularly after 10 days, whereas the growth of *G. trabeum* was only slightly affected (Table 1). The two fungi grew intertwined without forming visible inhibition zones, pigmentation or demarcation lines at the contact interface between both mycelia (Fig. 1). The qualitative interaction pattern was similar on MEA or MDT media. Overall, comparable mycelial densities were observed between monocultures and co-cultures of both fungi (Fig. 1).

**Table 1:** Mycelial growth of *B. adusta* and *G. trabeum* in mono- and co-cultures on MEA and MDT media

MEA			MDT				
			Diameter of colonies (cm)				
Fungal strain	Culture condition	2 days		10 days	2 days		10 days
*B. adusta*	Monoculture	2.20 ± 0.10		9.80 ± 0.10	3.33 ± 0.11		11.66 ± 0.47
*G. trabeum*	Monoculture	1.20 ± 0.17		5.33 ± 0.32	1.17 ± 0.15		5.03 ± 0.49
*B. adusta*	Co-culture	1.67 ± 0.55		7.30 ± 0.46	3.27 ± 0.06		9.60 ± 0.26
*G. trabeum*	Co-culture	1.03 ± 0.06		4.17 ± 0.35	1.17 ± 0.21		5.57 ± 1.00

### Qualitative enzyme activity on solid media

**Fig. 2:**
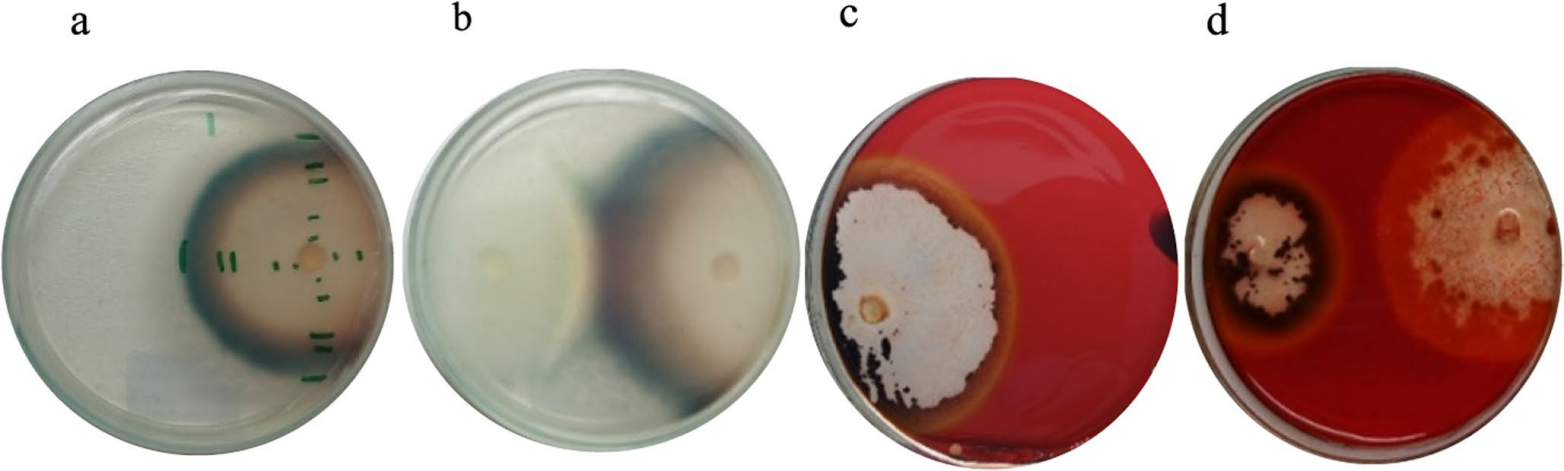
Plates with ABTS (0.02%) showing positive oxidation by monoculture (**a**) and co-culture (**b**) of *B. adusta*, and plates with CMC (1%) showing positive hydrolysis by monocultures (**c**) and co-culture (**d**) of *G. trabeum*, after 7 days of incubation at 25 °C. In the plates, the inoculum on the left corresponds to *G. trabeum* and the one on the right to *B. adusta*

ABTS oxidation was detected in plates inoculated with *B. adusta* in both monoculture (Fig. 2a) and the co-culture (Fig. 2b), as evidenced by the formation of a blue-green halo surrounding the colonies. The intensity and extension of the oxidation halo were higher in co-cultures of *B. adusta* with *G. trabeum* (Fig. 2b). As expected, *G. trabeum* did not exhibit ABTS oxidation when grown in monoculture (results not shown) or in co-culture, despite displaying mycelial growth comparable to *B. adusta* on ABTS- containing plates. On the other hand, *G. trabeum* produced a well-defined hydrolysis halo on the plates containing CMC in both monoculture and co-culture (Fig. 2c), confirming the production of extracellular cellulolytic activity. *B. adusta* formed dense, light-colored mycelium on these plates without hydrolysis halos in both monoculture (results not shown) and co-culture (Fig. 2d), which is consistent with its primary ligninolytic capability.

### Lignocellulolytic enzyme production in liquid media

**Fig. 3:**
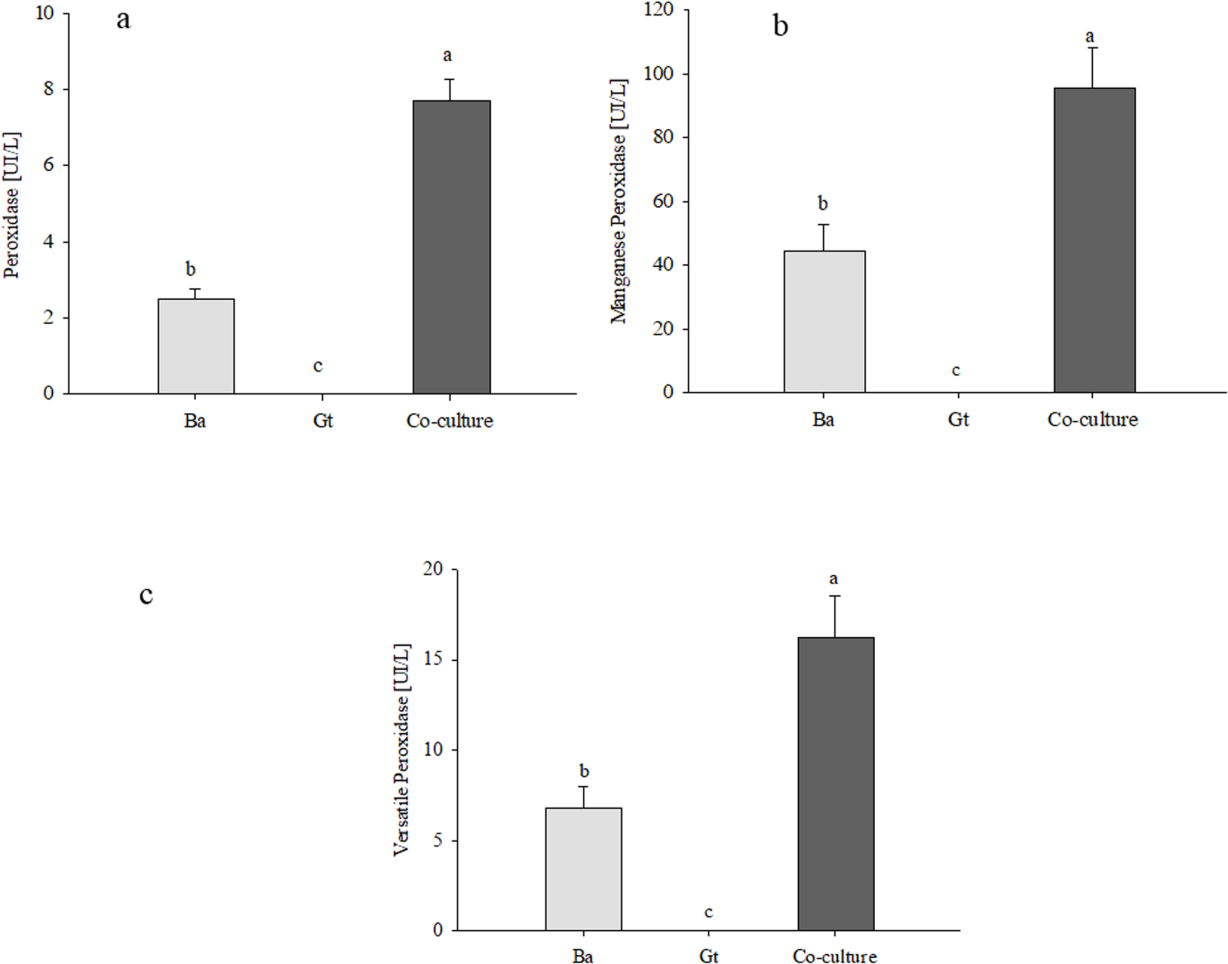
Oxidative enzymatic activities of peroxidase (**a**), manganese peroxidase (**b**), and versatile peroxidase (**c**), determined in monocultures and co-culture of *B. adusta* (Ba) and *G. trabeum* (Gt) after 10 days of incubation in liquid MDT medium supplemented with 1% wheat bran. Fungal filtrate broths were concentrated fivefold before analysis. Values correspond to the mean of three replicates ± standard deviation. Different letters above the bars indicate significant differences between treatments (p < 0.05)

Quantitative assays in liquid MDT medium showed that *B. adusta* predominantly produced peroxidase activity, including manganese peroxidase (MnP) and versatile peroxidase (VP), while no laccase activity was detected (Fig. 3). In co-culture, total peroxidase activity increased approximately threefold compared to the *B. adusta* monoculture (Fig. 3a). Manganese peroxidase (MnP) and versatile peroxidase (VP) increased by approximately 2.3-fold in co-culture relative to monoculture (Fig. 3b and c). No oxidative enzyme activities (peroxidase, MnP or VP) were detected in *G. trabeum* monocultures.

In contrast to the oxidative activities, hydrolytic enzyme activities were detected in monocultures of both fungi. However, these activities were significantly higher in *G. trabeum* cultures than in *B. adusta* cultures (Fig. 3). In co-culture, cellulolytic and hemicellulolytic activities were significantly reduced compared to monocultures. Particularly, endoglucanase, xylanase and β-xylosidase activities were severely suppressed (Fig. 3b-d), while β-glucosidase activity was least affected, decreasing by approximately 35% relative the highest monoculture value (Fig. 3a).

The highest soluble protein concentration was detected in the co-culture, however the value achieved was lower than the sum of the protein concentration measured in the two monocultures individually (Table 2). After 10 days of cultivation, a decrease of one unit in pH was observed only in the *G. trabeum* monoculture. The pH values of *B. adusta* extracts in monoculture and co-culture remained close to the initial pH of 5 (Table 2).

**Table 2:** Soluble protein content and pH of enzymatic extracts from monocultures and co-culture of *B. adusta* and *G. trabeum*

Culture condition	Protein (mg/mL)	pH
*B. adusta*	0.049 ± 0.002	4.65 ± 0.02
*G. trabeum*	0.036 ± 0.009	3.98 ± 0.34
Co-culture	0.065 ± 0.003	4.98 ± 0.03

The initial pH of MDT medium was adjusted to 5.0

### Characterization of protein and enzyme profiles in co-culture

**Fig. 4:**
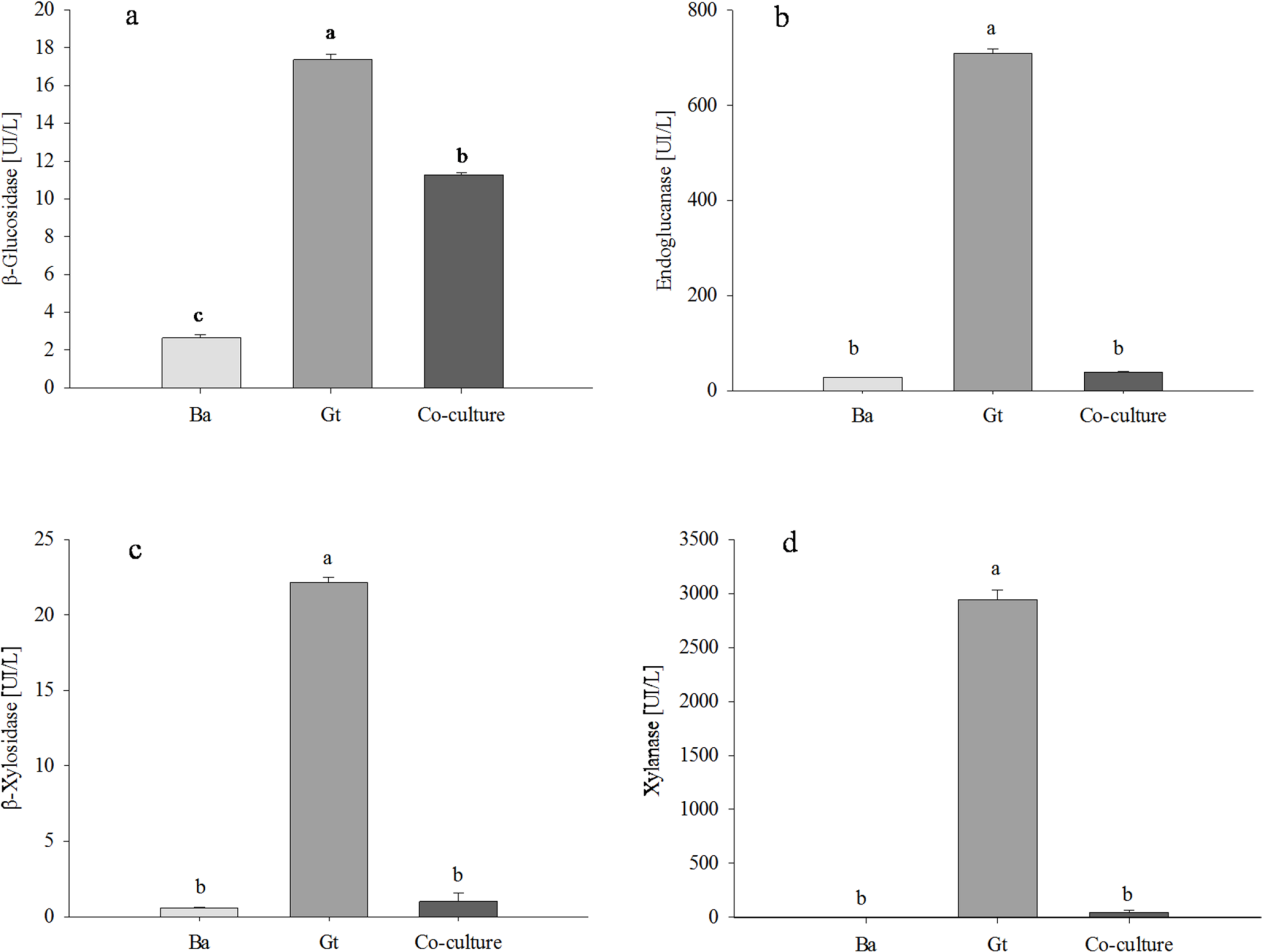
Hydrolytic enzymatic activities of β-glucosidase (**a**), β-xylosidase (**b**), endoglucanase (**c**), and xylanase (**d**) determined in monocultures and co-culture of *B. adusta* (Ba) and *G. trabeum *(Gt) after 10 days of incubation in liquid MDT medium supplemented with 1% wheat bran. Fungal filtrate broths were concentrated fivefold before analysis. Values correspond to the mean of three replicates ± standard deviation. Different letters above the bars indicate significant differences between treatments (p < 0.05)

Electrophoresis was performed under non-denaturing conditions (native PAGE) to analyze the protein profiles in the fungal extracts from monocultures and co-cultures, and a zymogram was used to detect peroxidase activity. Native PAGE revealed distinct protein profiles among the cultures. The *B. adusta* extract displayed multiple protein bands in the 20–66 kDa range, while the *G. trabeum* extract showed fewer bands, predominantly between 40 and 90 kDa (Fig. 4a). Co-culture extracts exhibited a protein profile similar to that of *B. adusta*, with the absence of bands below 30 kDa. This suggests the suppression of specific *B. adusta* proteins and the intensification of bands near 35 kDa. Additionally, the smaller (~ 40 kDa) band present in the *G. trabeum* monoculture was absent in the co-culture (Fig. 4a).

Zymogram analysis confirmed the ABTS-oxidizing activity in *B. adusta* and co-culture extracts at approximately ~ 35 kDa, with higher signal intensity in the co-culture. No ABTS oxidation was detected in *G. trabeum* extracts (Fig. 4b).

Ion-exchange chromatography on DEAE-Sepharose efficiently separated the unbound endoglucanases (peak 1) from the bound peroxidase (peak 2), in both monocultures and co-cultures (Supplementary Fig. 2). *G. trabeum* extracts showed a high peak 1 of endoglucanase activity, which was also present in *B. adusta* extracts. Conversely, *B. adusta* and co-culture exhibited a strong peak 2 of peroxidase activity, which was most intense in co-culture and absent in *G. trabeum*.

### Remazol brilliant blue R (RBBR) decolorization

**Fig. 5:**
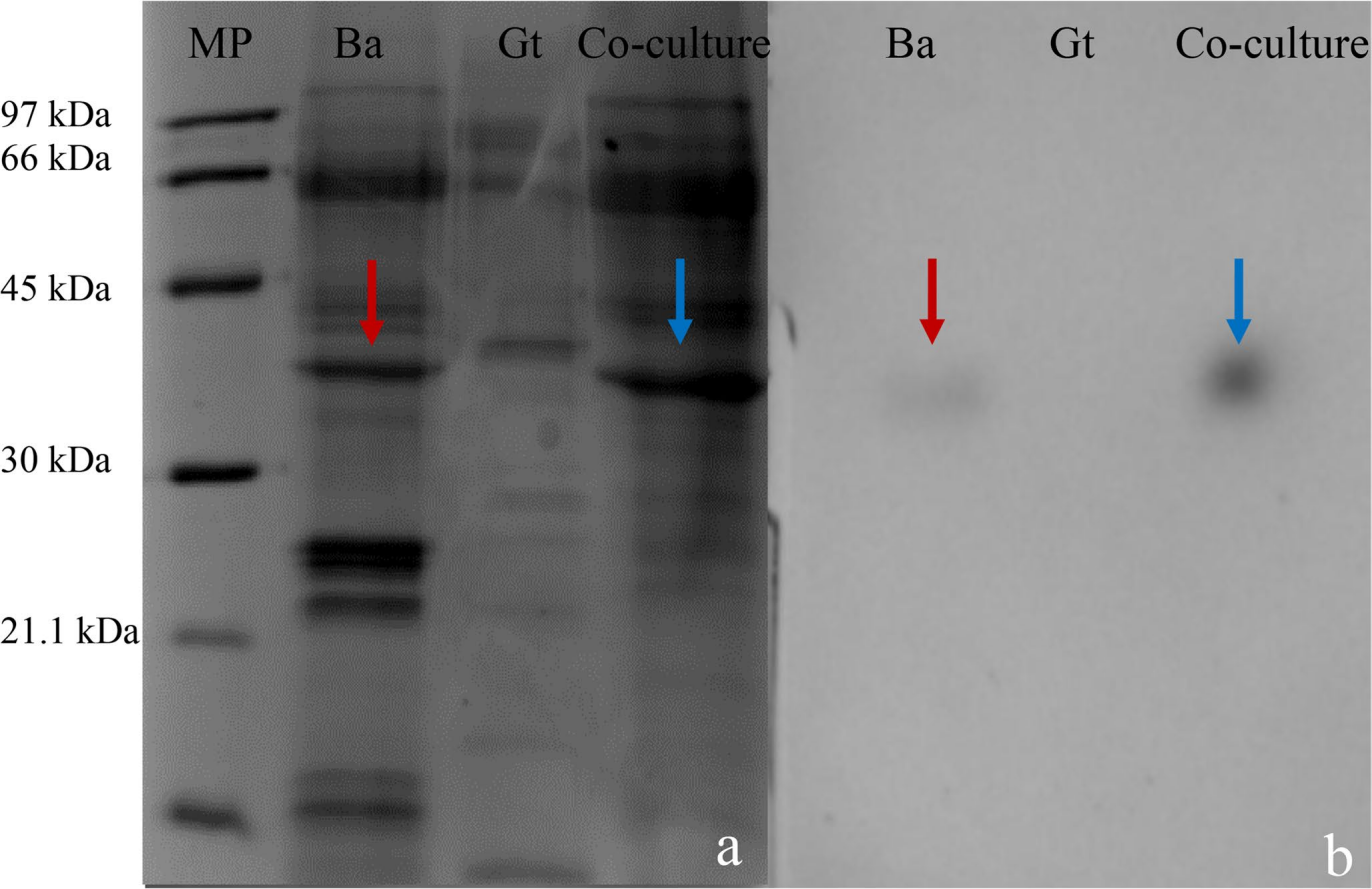
Native PAGE electrophoresis of extracts of *B. adusta* (Ba) and *G. trabeum* (Gt) and from the co-culture (Ba–Gt). (**a**) Extracellular protein profiles of monocultures and co-culture, and (**b**) zymogram for peroxidase detection using 2 mM ABTS and 20 mM H_2_O_2_. MP- molecular weight marker (kDa). Arrows indicate the protein bands of *B. adusta* (red) and co-culture (blue) that correspond to peroxidase activity bands. Fungal extracts were concentrated 20X prior to analysis

**Fig. 6:**
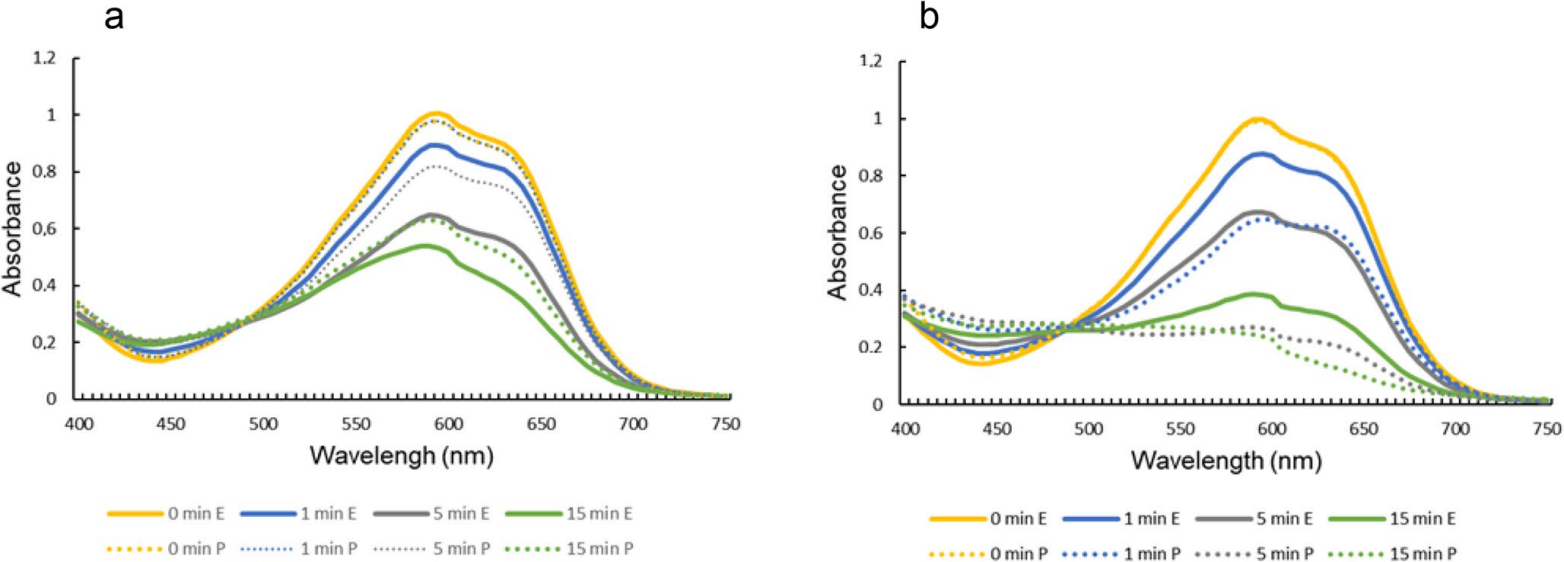
Visible spectra of RBBR (100 mg/L) at different treatment times (min) by *B. adusta* monoculture (**a**) and *B. adusta* - *G. trabeum* co-culture (**b**) using a standardized extract containing 0.02 U of peroxidase (**E**) or standardized extract containing 10 µg of protein (**P**)

RBBR (100 mg L⁻¹) decolorization assays revealed that co-culture extracts exhibited superior oxidative capacity compared to *B. adusta* monoculture extracts. When the dye treatment was standardized for peroxidase activity (0.02 U), both *B. adusta* and co-culture extracts initially decolorized RBBR at similar rates. However, only the co-culture extract maintained continuous decolorization, reaching 86% after 60 min (Figs. 5 and 6a). By contrast, *B. adusta* monocultures reached a maximum discoloration of only 43% within 10 min, and after that, the discoloration remained constant.

**Fig. 7:**
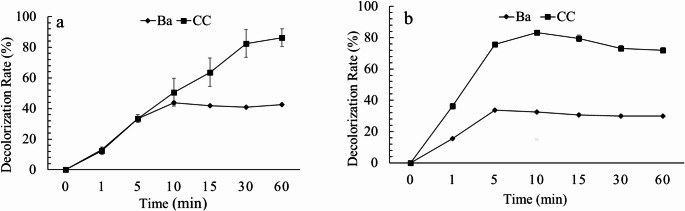
Decolorization rate of RBBR (%) by monoculture of *B. adusta* (Ba) and co-culture (CC), standardized to an extract containing 0.02 U of peroxidase (**a**) and to an extract containing 10 µg of protein (**b**), at different incubation times, pH 5, 2 mM H2O2, and 30 °C

When the RBBR treatments were standardized for protein content (10 µg), *G. trabeum* showed no decolorization activity, while *B. adusta* achieved a maximum decolorization of approximately 40% at 10 min. The co-culture extract achieved a maximal decolorization of 83% within the same time (Figs. 5 and 7b), after that, the decolorization remained approximately constant for both monocultures and co-culture until 60 min. Specifically, the co-culture exhibited higher specific activities for all enzymes evaluated (peroxidase: 6.3 U mg⁻¹; VP: 0.27 U mg⁻¹; MnP: 0.94 U mg⁻¹) than the monoculture (peroxidase: 3.5 U mg⁻¹; VP: 0 U mg⁻¹; MnP: 0.31 U mg⁻¹).

## Discussion

Co-cultivation assays demonstrated that the interaction between the white-rot fungus *Bjerkandera adusta* and the brown-rot fungus *Gloeophyllum trabeum* results in marked metabolic reprogramming. This interaction alters both morphology and growth kinetics of involved mycelia (Zoglowek et al. 2016; Pinheiro et al. 2021). Initial solid-medium assays indicated partial compatibility between the species. This was evidenced by interconnected mycelial growth and the absence of a visible antagonistic barrier (Molla et al. 2001). No pigmentation or clear demarcation line was observed at the contact interface, which suggests a non-antagonistic interaction and possible cooperation (Boddy and Hiscox 2016). Fungal growth was only slightly reduced in co-culture compared to monocultures. The pattern of mycelial interactions was not affected by the composition of the medium (MEA or MDT). Such non-antagonistic interactions have been reported for related fungal systems. These are often associated with metabolic adjustments rather than direct growth inhibition, allowing coexistence and functional complementarity between interacting species.

The increased ABTS oxidation halo in plate- based solid medium observed in co-culture compared to *B. adusta* monoculture suggests that interspecific interaction stimulates oxidative enzyme production. This effect is likely triggered by the proximity of *G. trabeum*. This response is consistent with previous studies showing that co-cultivation interactions can act as ecological cues that trigger ligninolytic enzyme expression, as part of defense, competition, or adaptive strategy (Cui et al. 2021; Naseema Rasheed et al. 2023). In contrast, *G. trabeum* monocultures showed no ABTS oxidation in line with the absence of ligninolytic activity in brown-rot fungi (Goodell et al. 2020).

Complementary to the plate-based detection of oxidative enzyme activity, cellulose hydrolysis assays on CMC agar revealed functional differentiation between the two fungi. *G. trabeum* produced a well-defined hydrolysis halo in both monoculture and co-culture, confirming cellulolytic activity (Miotto et al. 2014). Although mycelial growth of *G. trabeum* was slightly reduced in the presence of *B. adusta*, hydrolysis remained unchanged. This indicates that cellulase secretion was maintained. In contrast, *B. adusta* formed a dense, light-colored mycelium on CMC medium, but did not produce a detectable hydrolysis halo, consistent with its primarily ligninolytic metabolism (Sugawara and Sugano 2023). Plate assays for enzyme detection are qualitative but provide useful observations about enzyme secretion that cannot be made in a liquid medium, such as identification of the enzyme-producing organism during co-culture.

The co-cultivation of *B. adusta* and *G. trabeum* in submerged fermentation experiments further confirmed changes in enzyme secretion patterns and enhanced significantly (*p* < 0.05) the oxidative activities. These results corroborate reports that co-cultures can overcome metabolic limitations of monocultures, activating silent gene clusters and the emergence of novel enzymatic pathways (Ming et al. 2019; Intasit et al. 2021; Peláez et al. 2022; Soares et al. 2022; Kim 2023).

In monoculture, *B. adusta* exhibited high oxidative activity, mainly due to the secretion of peroxidases (MnP and VP). Although *B. adusta* contains laccase-encoding genes, no laccase activity was detected under the conditions evaluated. Laccase expression in *B. adusta* depends strongly on culture conditions and strain variability. In several studies, its is low or only detected under specific nutritional or environmental conditions (Belcarz et al. 2005; Heinfling et al. 1998).

Co-cultivation increased peroxidase activities by two- to threefold, indicating that interspecific interactions can amplify oxidative capability beyond levels achieved in monoculture. Similar effects have been reported in other white-rot fungal co-cultures systems, suggesting that this response is not species-specific but reflects a broader regulatory phenomenon (Sugano et al. 2021; Cui et al. 2023). The stimulation of peroxidase activity in the presence of *G. trabeum* may reflect stress -related signaling or competition for nutrients and space, both of which are known to activate ligninolytic gene expression in white-rot fungi (Detain et al. 2022; Tironi et al. 2024). From an ecological perspective, such responses may confer an advantage to white-rot fungi by accelerating oxidative modification of the substrate, improving access to structurally complex carbon sources.

*G. trabeum* monocultures produced significantly higher cellulolytic and hemicellulolytic activities than *B. adusta*, in agreement with the brown-rot decay mechanism based on radical-mediated polysaccharide depolymerization (Qi et al. 2023). In co-culture, hydrolytic activities were significantly suppressed, indicating that interspecific interactions constrain on carbohydrate-degrading enzyme systems. This selective suppression may arise from antagonistic metabolic interactions, resource competition, oxygen limitation, or localized pH changes (Tripathi et al. 2012; Tironi et al. 2024; Saez et al. 2025). Among the enzymes evaluated, β-glucosidase activity was less affected than endoglucanase and xylanase, indicating that co-culture conditions differentially modulate hydrolytic enzyme secretion. All enzymatic activities were measured at 10 days. Therefore, a delay in enzyme production cannot be excluded.

Overall, co-cultivation shifted the enzymatic balance toward oxidative metabolism. Peroxidases activities increased, while hydrolytic enzymes decreased. Similar patterns have been reported in mixed white-rot fungal systems such as *Lenzites betulina* and *Trametes versicolor* (Cui et al. 2023), reinforcing that interspecific interactions can selectively activate oxidative pathways. These findings support the concept that co-cultivation can induce silent oxidative pathways and upregulate enzyme expression involved in complex substrate degradation (Selegato and Castro-Gamboa 2023).

Protein profiles and zymograms provide additional evidence of metabolic reprograming in *B. adusta* during co-cultivation. The disappearance of low-molecular-weight bands and increased peroxidase signal intensity around 35 kDa suggest changes in enzyme expression or post-translational modification. These alterations may be linked to fungal recognition mechanisms, potentially mediated by surface glycoepitopes, which have been shown to activate defense-related or competitive responses in fungi (John et al. 2024). Ion-exchange chromatography further revealed differences in peroxidase properties between monoculture and co-culture extracts, suggesting that co-cultivation may influence enzyme isoform composition. Peroxidase activity was exclusive to *B. adusta* and co-culture extracts, showing higher affinity in co-culture. Post-translational modifications may improve enzyme stability or activity (Dullah et al. 2021; González et al. 2021).

The metabolic complementarity between *B. adusta* (white-rot) and *G. trabeum* (brown-rot) highlights the potential of combining decay mechanisms for lignocellulose valorization and bioremediation of xenobiotic compounds. Co-culture can generate multifunctional enzyme cocktails, discover novel metabolites, and improve degradation of recalcitrant pollutants (Peng et al. 2021; Peláez et al. 2021; Yu et al. 2021; Prabhu et al. 2022).

The functional consequences of this metabolic complementarity were clearly demonstrated in dye decolorization assays. Despite normalization of peroxidase units, co-culture extracts achieved nearly 90% RBBR degradation in 60 min, while *B. adusta* reached ~ 40%. This suggests the involvement of additional enzymes or metabolites, beyond those evaluated here. *G. trabeum* monocultures did not decolorize RBBR, however, in co-culture, it may enhance the degradation through multiple mechanisms. These include acting as an elicitor of enzyme production in *B. adusta*, promoting non-enzymatic oxidation via Fenton chemistry and hydroxyl radical generation, and/or producing auxiliary enzymes that support oxidative processes (Jensen et al. 2001; Umezawa et al. 2020).

The enhanced performance observed in the co-culture system may be partially explained by the enzymatic repertoire of both fungi, particularly the presence of genes encoding proteins from the auxiliary activity family 9 (AA9). Both *B. adusta* and *G. trabeum* harbor genes associated with this family, which includes lytic polysaccharide monooxygenases (LPMOs) known to play a key role in the oxidative cleavage of recalcitrant polysaccharides (Kojima et al. 2016; Hegnar et al. 2019). Previous genomic and biochemical studies have shown that *G. trabeum* possesses a diverse set of genes encoding accessory enzymes involved in lignocellulose deconstruction. Notably, the genome of *G. trabeum* contains at least 26 AA9-encoding genes, highlighting its potential for oxidative biomass degradation. Among these, two AA9 enzymes have been successfully cloned and secreted, demonstrating their functional relevance. Moreover, experimental evidence indicates that *G. trabeum* produces a broader diversity of AA9 enzymes compared to other brown-rot fungi, which may contribute to its efficiency in degrading plant cell walls (Umezawa et al. 2020).

Supporting this notion, protein fractions obtained from *G. trabeum* cultures in our laboratory were shown to enhance the hydrolysis of wheat straw when combined with cellulases. Proteomic analysis of these fractions revealed that the most abundant proteins were the xylanase GtXyn10A and a lytic polysaccharide monooxygenase (GtLPMO) (unpublished results). The presence of these enzymes suggests a synergistic mechanism in which oxidative cleavage by AA9 enzymes increases substrate accessibility, thereby improving the action of hydrolytic enzymes such as cellulases and hemicellulases.

Taken together, these findings indicate that AA9 enzymes, particularly those produced by *G. trabeum*, play a significant role in enhancing lignocellulosic biomass conversion. In the context of co-cultivation with *B. adusta*, this enzymatic complementarity may contribute to improved degradation efficiency, potentially through synergistic interactions between oxidative and hydrolytic systems. Such interactions highlight the relevance of co-culture strategies for optimizing enzyme systems aimed at biomass valorization.

Overall, co-cultivation of white- and brown-rot fungi promotes oxidative metabolism and suppress hydrolytic pathways. Such rebalancing of enzymatic activities underscores the potential of fungal co-culture systems for lignin degradation, offering a low-cost and synergistic route for biotechnological applications. The integration of complementary enzymatic and probably non-enzymatic systems not only improves degradation of recalcitrant dyes but also opens opportunities for lignocellulosic biomass valorization, pollutant bioremediation, and discovery of novel bioactive metabolites (Peláez et al. 2021; Yu et al. 2021; Prabhu et al. 2022). Furthermore, co-cultivation is a promising platform for expanding the available enzymatic and metabolic diversity for generating robust oxidative enzyme cocktails.

## Supplementary Information

Below is the link to the electronic supplementary material.

Supplementary Material 1 (DOCX 1.38 MB)

## Data Availability

The study’s supporting data can be found in the manuscript. For access to the raw data, please reach out to the corresponding author with a reasonable request.
