# Supplementary material for: Changes in the enzyme profile of the white-rot fungus Bjerkandera adusta in co-culture with the brown-rot fungus Gloeophyllum trabeum and its potential for dye decolorization
Source: World J Microbiol Biotechnol. 2026 May 12;42(6):281. doi: 10.1007/s11274-026-05018-5 (PMC13167839; doi:10.1007/s11274-026-05018-5)

Fig. S1. Diagram illustrating the inoculation of coculture on Petri dishes, with one agar-mycelium disc of each fungus on the same plate, 5 cm apart. Gt = *Gloeophyllum trabeum*, Ba = *Bjerkandera adusta*.


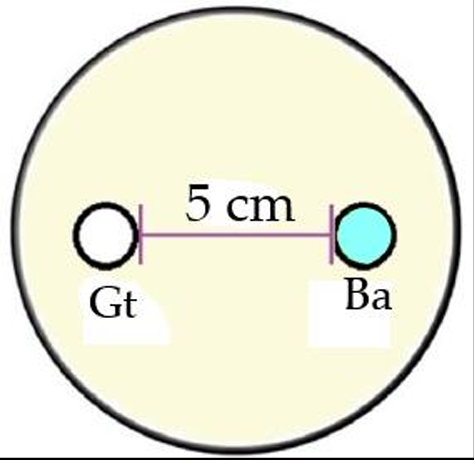


Fig. S2. Protein profile of fungal extracts purified by ion-exchange chromatography using an FPLC system. (a) *B. adusta* monoculture, (b) *G. trabeum* monoculture, (c) co-culture of *B.adusta* and *G. trabeum*. (1) Peak with endoglucanase activity; (2) peak with peroxidase activity. Separation was performed on a 10 mL DEAE-Sepharose CL6B anion-exchange column and eluted with a 500 mM NaCl gradient. Fungal extracts were concentrated fivefold prior to analysis.


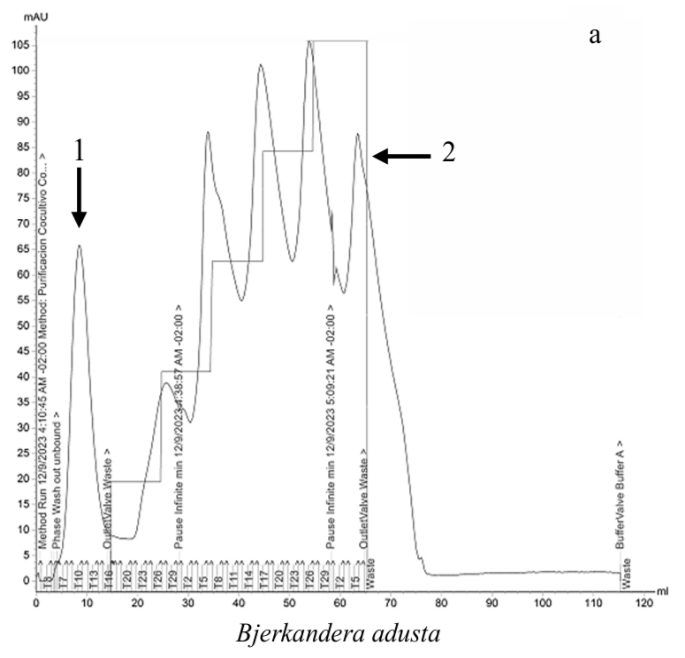

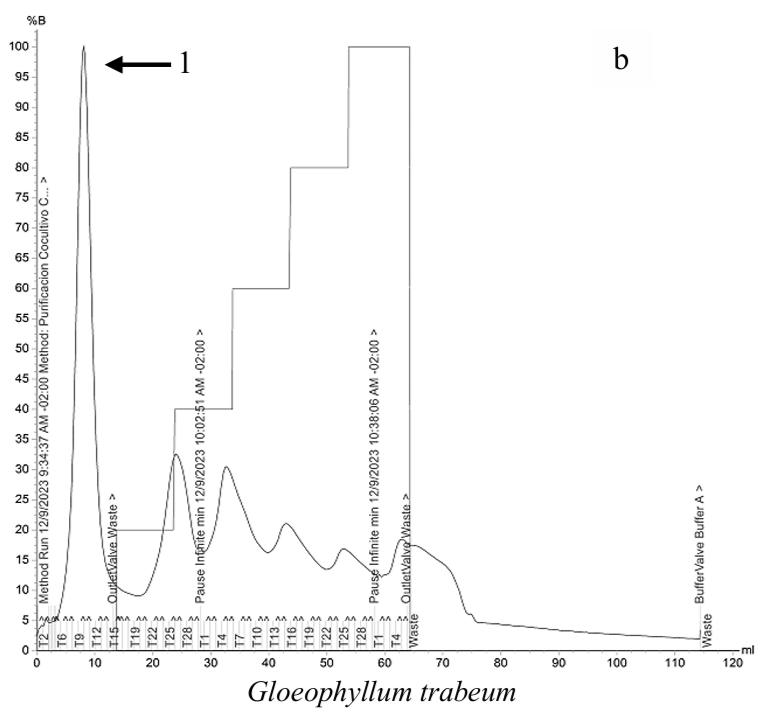

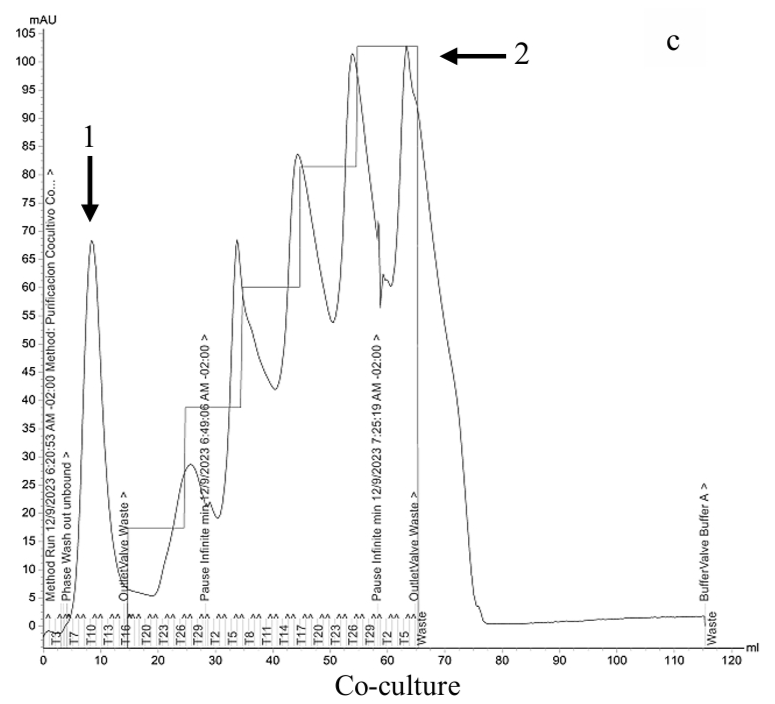

Supplement: Supplementary file 1 — Supplementary Material 1 (DOCX 1.38 MB) [file 11274_2026_5018_MOESM1_ESM.docx]
